# Xylanase and phytase as modulators of gut microbiota and phytate degradation in wheat-based diets for meat quail

**DOI:** 10.1016/j.psj.2026.106935

**Published:** 2026-04-15

**Authors:** Iva Carla de Barros Ayres, Adiel Vieira de Lima, Aline Beatriz Rodrigues, Paloma Eduarda Lopes de Souza, Carlos Henrique do Nascimento, Marcos Aurelio Victor de Assunção, Danilo Vargas Gonçalves Vieira, Alexandre Barbosa de Brito, Apolônio Gomes Ribeiro, Ricardo Romão Guerra, Thácyla Beatriz Duarte Correia, José de Arimatéia de Freitas Pinto, Fernando Guilherme Perazzo Costa, Lucas Rannier Ribeiro Antonino Carvalho, Matheus Ramalho de Lima

**Affiliations:** a Department of Animal Science, Federal University of Paraíba, Areia, Paraíba, Brazil; b Department of Animal Sciences, Federal Rural University of the Semi-Arid Region, Mossoró, Rio Grande do Norte, Brazil; c Federal University of Northern Tocantins, School of Veterinary Medicine and Animal Science, Araguaína, Tocantins, Brazil; d Global Director of ABVista, 3 Woodstock Court, Blenheim Road Marlborough Business ParkSN84AN, Marlborough, UK; e Department of Veterinary Sciences, Federal University of Paraíba, Areia, Paraíba, Brazil; f Department of Physiology and Pharmacology, Karolinska Institutet, Biomedicum 5B, Solnavägen 9, S-171 77, Stockholm, Sweden

**Keywords:** Nutritional basis, Enzymes, Firmicutes

## Abstract

This study evaluated the interactive effects of xylanase and phytase in corn- or wheat-based diets on growth performance, phytate degradation, digesta pH, and gut microbiota of meat-type quail. A total of 224 European quail were assigned to a 2 × 2 × 2 factorial arrangement with two basal diets (corn–soybean meal or wheat–soybean meal), two xylanase levels (0 or 16,000 BXU/kg), and two phytase levels (0 or 2,000 FTU/kg). Growth performance was evaluated from 7 to 42 d of age, and carcass traits, intestinal pH, inositol phosphate (InsP) concentrations, and gut microbiota were assessed at 42 d. Birds fed wheat-based diets exhibited greater body weight gain and improved feed conversion compared with those fed corn-based diets (*P* < 0.05). Phytase and xylanase supplementation enhanced phytate degradation, reduced concentrations of higher-order inositol phosphates (InsP6–InsP4), and modified digesta pH (*P* < 0.05), with more pronounced effects in wheat-based diets. Alpha diversity analysis indicated no effect of treatments on bacterial richness (Chao1), whereas Shannon diversity differed markedly among treatments (*P* < 0.001), indicating changes in microbial evenness. Beta diversity analyses revealed a clear separation of microbial communities according to basal diet and enzyme supplementation (PERMANOVA, *P* = 0.001). Correlation network analysis demonstrated matrix-dependent reorganization of microbial interactions in response to phytase and xylanase. In conclusion, phytase and xylanase supplementation modulated gut microbial community structure and phytate degradation in a basal diet-dependent manner. Productive performance responses were influenced by interactions between enzyme supplementation and diet composition. These findings highlight the importance of enzyme–matrix interactions in shaping nutrient availability and intestinal microbial ecology in meat-type quail.

## Introduction

Wheat-based diets are widely used in poultry production due to their economic advantages and regional availability. However, their high content of soluble non-starch polysaccharides (NSP), particularly arabinoxylans, increases digesta viscosity, impairs nutrient diffusion, and reduces nutrient utilization efficiency. Elevated intestinal viscosity disrupts digesta flow and limits nutrient absorption, ultimately compromising growth performance in poultry (Nguyen et al., 2022).

Supplementation with exogenous enzymes, particularly xylanase and β-glucanase, has been extensively shown to mitigate these adverse effects by depolymerizing NSP, thereby reducing digesta viscosity and improving nutrient accessibility (Zhang et al., 2014). Xylanase-mediated cleavage of the arabinoxylan backbone decreases molecular weight and viscosity, facilitating improved nutrient diffusion and absorption. Moreover, the use of multi-activity enzymes containing glucanase and xylanase has been reported to improve growth performance and nitrogen utilization, particularly in wheat-based diets, by enhancing the breakdown of NSP (Godbout et al., 2024). The effectiveness of these enzymes is more pronounced in wheat-based diets compared with corn-based diets, highlighting the specific nutritional constraints associated with wheat-derived NSP (Godbout et al., 2024). Overall, while wheat-based diets present challenges due to their NSP content, strategic enzyme supplementation can significantly improve nutrient utilization and broiler performance, making these diets more viable in poultry production (Ayres et al., 2019).

Xylanase and phytase supplementation in animal diets has been shown to significantly enhance nutrient digestibility and growth performance by reducing digesta viscosity and improving nutrient accessibility. Xylanase works by breaking down non-starch polysaccharides, which decreases the viscosity of the digesta, thereby facilitating better nutrient absorption and promoting intestinal health. This reduction in viscosity allows for improved nutrient flow and absorption in the gut, leading to enhanced growth performance in animals such as nursery pigs and broiler chickens (Anwar et al., 2023). Phytase, on the other hand, hydrolyzes phytate (InsP6), which is a major antinutritional factor in animal feeds, thereby increasing the availability of phosphorus. This enzymatic action not only improves phosphorus digestibility but also reduces the need for inorganic phosphorus supplements, which can be both costly and environmentally detrimental (Moita, 2023; Walker et al., 2024). The combination of these enzymes has been shown to further enhance the digestibility of other nutrients, such as amino acids and energy, by breaking down complex feed components that would otherwise be inaccessible to the animal’s digestive enzymes (Widodo et al., 2018; Wu et al., 2004). Studies have demonstrated that the inclusion of xylanase and phytase in diets can lead to improvements in feed conversion ratios, body weight gain, and overall animal performance, while also contributing to better intestinal health by modulating the gut microbiota and reducing oxidative stress (Kayan et al., 2025; Moita and Kim, 2022). These benefits are particularly evident in wheat-based diets, where the presence of non-starch polysaccharides and phytate can significantly hinder nutrient availability (Anwar et al., 2023; Wu et al., 2004). Overall, the strategic use of xylanase and phytase in animal nutrition not only enhances growth performance and nutrient utilization but also offers economic and environmental advantages by reducing the reliance on inorganic phosphorus sources and improving feed efficiency (Moita, 2023).

Beyond their established effects on nutrient digestibility, diet composition and enzyme supplementation are increasingly recognized as major drivers of intestinal microbial ecology in poultry. Wheat-based diets, due to their higher content of fermentable non-starch polysaccharides, can markedly reshape microbial community structure. Xylanase supplementation not only reduces digesta viscosity but also generates xylo-oligosaccharides with potential prebiotic effects, whereas phytase-mediated phytate hydrolysis alters phosphorus availability and inositol phosphate profiles, with potential consequences for the intestinal ecosystem. However, limited information is available regarding how the combined use of phytase, and xylanase modulates gut microbiota in quail under different basal diet matrices.

Therefore, this study aimed to determine whether basal diet matrix (corn-soybean meal vs. wheat-soybean meal) and supplementation with phytase and xylanase, individually or in combination, modulate intestinal microbial community structure, diversity, and interaction networks in meat-type quail, and whether these microbial responses are coherent with changes in growth performance, carcass traits, digesta pH, and phytate (InsPs) hydrolysis. It was hypothesized that combined enzyme supplementation would enhance phytate degradation, improve productive performance, and promote a more functionally optimized intestinal microbial ecosystem, particularly in wheat-based diets.

## Materials and methods

The experimental trial was conducted at the Poultry Module of the Department of Animal Science, at the Center for Agricultural Sciences of the Federal University of Paraíba, Campus II, in Areia, Paraíba, Brazil. The experimental protocol N°. 7990100621 was approved by the Animal Use Ethics Committee of the Federal University of Paraíba (CEUA-UFPB).

### Birds, diets and experimental design

This study used 224 1-day-old European quails of mixed sex. The quails were randomly allocated into eight treatments, with seven replicates of four quails per replicate. The quails were fed a basal diet based on corn and soybean meal from 1 to 6 day-old, and from 7 to 42 days of age, the quails were fed experimental diets with use of xylanase and phytase (Table 1) balanced to meet the nutrient requirements for European quails (Silva et al., 2012). The initial body weight of the quails at the beginning of the experiment (7 days of age) was 49.933 ± 0.641 g. The quails were housed in cages (40×30×50 cm) with PVC feeder and automatic drinkers. The light program was 24 h. The temperature and humidity were as follows: from 7 to 21 days, 29.9 ± 0.91°C and 82.3 ± 3.81%, and from 22 to 42 days, 27.45 ± 0.98°C and 95.5 ± 1.83%.

**Table 1:** Feed and nutrient composition of the experimental diets.

Item		Corn + Soybean meal				Wheat + Soybean meal			
		T1	T2	T3	T4	T5	T6	T7	T8
Corn		61.826	61.826	61.826	61.826	-	-	-	-
Soybean meal		34.293	34.293	34.293	34.293	33.066	33.066	33.066	33.066
Wheat		-	-	-	-	49.634	49.634	49.634	49.634
Vegetable oil		0.738	0.738	0.738	0.738	14.570	14.570	14.570	14.570
Dicalcium phosphate		1.291	1.291	1.291	1.291	0.685	0.685	0.685	0.685
Limestone		0.867	0.867	0.867	0.867	1.129	1.129	1.129	1.129
DL-Methionine		0.309	0.309	0.309	0.309	0.308	0.308	0.308	0.308
Salt		0.380	0.380	0.380	0.380	0.387	0.387	0.387	0.387
L-Lysine		0.137	0.137	0.137	0.137	0.063	0.063	0.063	0.063
Vitamin premix^1^		0.080	0.080	0.080	0.080	0.080	0.080	0.080	0.080
Mineral premix^2^		0.050	0.050	0.050	0.050	0.050	0.050	0.050	0.050
Xylanase^3^			0.010		0.010		0.010		0.010
Phytase^4^				0.020	0.020			0.020	0.020
Washed sand		0.030	0.020	0.010	-	0.030	0.020	0.010	-
Total		100	100	100	100	100	100	100	100

Calculated composition		Corn + Soybean meal				Wheat + Soybean meal			
	T1	T2	T3	T4	T5	T6	T7	T8	
Crude protein	%	20.956	20.956	20.956	20.956	20.926	20.926	20.926	20.926
Crude Fiber	%	2.925	2.925	2.925	2.925	6.584	6.584	6.584	6.584
Calcium	%	0.750	0.750	0.750	0.750	0.750	0.750	0.750	0.750
Available Phosphorus	%	0.350	0.350	0.350	0.350	0.350	0.350	0.350	0.350
Metabolizable Energy	kcal/kg	2,950	2,950	2,950	2,950	2,950	2,950	2,950	2,950
Digestible Arginine	%	1.318	1.318	1.318	1.318	1.318	1.318	1.318	1.318
Isoleucine	%	0.820	0.820	0.820	0.820	0.820	0.820	0.820	0.820
Lysine	%	1.140	1.140	1.140	1.140	1.140	1.140	1.140	1.140
Methionine	%	0.606	0.606	0.606	0.606	0.589	0.589	0.589	0.589
Methionine + Cystine	%	0.890	0.890	0.890	0.890	0.890	0.890	0.890	0.890
Threonine	%	0.707	0.707	0.707	0.707	0.707	0.707	0.707	0.707
Tryptophan	%	0.232	0.232	0.232	0.232	0.232	0.232	0.232	0.232
Valine	%	0.880	0.880	0.880	0.880	0.880	0.880	0.880	0.880
Sodium	%	0.170	0.170	0.170	0.170	0.170	0.170	0.170	0.170
Chloride	%	0.274	0.274	0.274	0.274	0.274	0.274	0.274	0.274
Potassium	%	0.801	0.801	0.801	0.801	1.116	1.116	1.116	1.116

1 Vitamin premix (per kg of product): Vit. A – 15,000 IU; Vit. D₃ – 1,500,000 IU; Vit. E – 15,000 IU; Vit. B₁ – 2.0 g; Vit. B₂ – 4.0 g; Vit. B₆ – 3.0 g; Vit. B₁₂ – 0.015 g; niacin – 25 g; pantothenic acid – 10 g; Vit. K₃ – 3.0 g; folic acid – 1.0 g.

2 Mineral premix (per kg of product): Mn – 60 g; Fe – 80 g; Zn – 50 g; Cu – 10 g; Co – 2 g; I – 1 g; Se – 250 mg.

3 Enzymes: Econase XT (AB Vista); ^4^Quantum Blue (AB Vista).

Eight treatment groups in factorial design arrangement with 2 × 2 × 2; two diets based on corn and soybean meal, and wheat and soybean meal; two xylanase doses, 0 and 16000BXU (endo‐1,4‐β‐xylanase); two phytase doses, 0 and 2000FTU (6‐phytase). Feed and water were offered ad libitum to quails during the experimental period. The chemical composition of the diets was determined based on the methods of the Association of Official Analytical Chemists (AOAC, 1997), and the analyzed composition of the experimental diets is presented in Table 2.

**Table 2:** Analyzed composition of the experimental diets.

Analyzed composition	T1	T2	T3	T4	T5	T6	T7	T8
Crude protein	19.25	20.11	19.45	19.65	20.78	20.01	21.43	20.59
Crude Fiber	3.02	2.98	3.34	3.07	7.03	7.39	7.12	7.05
Calcium	0.73	0.75	0.76	0.77	0.76	0.75	0.76	0.73
Phosphorus	0.37	0.36	0.36	0.37	0.37	0.38	0.37	0.38
Metabolizable Energy	2986.2	2897.4	2964.3	2970.4	2984.2	2895.7	2954.8	2949.5
Lysine	1.2	1.2	1.2	1.2	1.2	1.2	1.2	1.2
Methionine	0.6	0.6	0.6	0.6	0.6	0.6	0.6	0.6
Threonine	0.7	0.7	0.7	0.7	0.7	0.7	0.7	0.7
Valine	0.9	0.9	0.9	0.9	0.9	0.9	0.9	0.9
Tryptophan	0.3	0.3	0.3	0.3	0.3	0.3	0.3	0.3
Xylanase	-	15,680	-	15,840	-	15,680	-	15,840
Phytase			1,960	1,980			1,960	1,980

Phytase added to the experimental diets was from *Escherichia coli* (*E. coli*) produced in *Thricoderma reesei* (Quantum Blue, AB Vista, Marlborough, UK; 5,000 FTU/g). Xylanase added to the experimental diets was a β−1,4 endo-xylanase produced by *Trichoderma reesei* (Econase XT 25P, AB Vista, Marlborough, UK; 160,000 BXU/g).

Enzyme activities in the experimental diets were determined by a commercial laboratory (CBO Análises, Brazil) to verify the recovery of supplemented enzymes. The analyzed values confirmed adequate recovery, corresponding to approximately 98% of the expected levels when enzymes were added individually (xylanase: 15,680 BXU/kg; phytase: 1,960 FTU/kg) and 99% when supplemented in combination (xylanase: 15,840 BXU/kg; phytase: 1,980 FTU/kg). Enzyme activities are expressed as BXU (birch xylan units), defined as the amount of enzyme that releases reducing sugars equivalent to 1 nmol of xylose per second under assay conditions, and FTU (phytase units), defined as the amount of enzyme that releases 1 μmol of inorganic phosphorus per minute from sodium phytate at pH 5.5 and 37°C. The analyzed enzyme activities are presented in Table 2.

### Performance and carcass analysis

For the evaluation of bird performance, body weight and feed intake were recorded at the replicate (cage) level on a weekly basis. Data were then expressed on a per-bird basis by dividing the total values by the number of birds in each replicate. Body weight gain was determined by calculating the differences in body weight between consecutive weeks. Feed conversion ratio (g feed intake/g body weight gain) was calculated by dividing feed intake by body weight gain on a weekly basis.

At 42 days of age, the birds were subjected to an 8-hour fasting period, except for those designated for intestinal content collection for microbiome analysis. Subsequently, ten birds from each treatment were randomly selected across replicates and euthanized by cervical dislocation for the evaluation of carcass yield and small intestine length. Similarly, at the end of the study, 80 quails were randomly selected across replicates, and the small intestine contents were immediately collected and placed into sterile Falcon tubes for microbiota enumeration and analysis.

### pH and Phytate (myo-inositol)

At the end of the experiment, digesta pH was analyzed, and samples of small intestinal digesta were collected for phytate evaluation. Two milliliter aliquots of the supernatants were diluted with water to give a final volume of 60 mL. The entire solution was applied to a column (0.7 × 15 cm) containing 0.5 g of AG 1-X4 200−400-mesh resin. The column was washed with 25 mL of water and 25 mL of 25 mM HCl. Then myo-inositol phosphates were eluted with 25 mL of 2 M HCl. The eluates obtained were concentrated in a vacuum evaporator and dissolved in 1 mL of water. Then 20 μL of the samples was chromatographed on Ultrasep ES 100 RP18 (2 × 250 mm). The column was run at 45°C and 0.2 mL min-1 of an eluant consisting of formic acid/methanol/water/tetrabutylammonium hydroxide (44:56:5:1.5 v/v), pH 4.25, as described by Sandberg and Ahderinne.

### Small intestine microbiota analysis

The bacteria were identified via high-throughput sequencing of 16S rRNA V3/V4 region with a proprietary protocol (Neoprospecta Microbiome Technologies, Brazil). The amplification of the 16S rRNA V3/V4 region was carried out using the 341F (CCTACGGGRSGCAGCAG) and 806R (GGACTACHVGGGTWTCTAAT) primers. The 16S rRNA libraries were sequenced using the MiSeq Sequencing System (Illumina Inc., USA) with the V2 kit, 300 Cycles, single-end sequencing. The sequences were analyzed using a proprietary pipeline (Neoprospecta Microbiome Technologies, Brazil).

Briefly, all the reads were individually submitted to a quality filter, based on the sum of the DNA bases probabilities errors, allowing a maximum of 1% of accumulated errors. Subsequently, the DNA sequences corresponding to the Illumina adapters were removed. The resulting sequences that presented 100% identity were clustered and were used for taxonomic identification, using an accurate 16S rRNA sequences database (NeoRef, Neoprospecta Microbiome Technologies, Brazil).

### Statistical analysis

All zootechnical performance, carcass traits, and physicochemical parameters were analysed using analysis of variance (ANOVA) through the General Linear Model (GLM) procedure of SAS software (SAS Institute Inc., Cary, NC, USA). When significant effects were detected, means were compared using Tukey’s multiple range test. Differences were considered statistically significant at *P* < 0.05.

Microbial alpha diversity indices (Chao1 and Shannon) were analyzed using Welch’s analysis of variance to account for potential heteroscedasticity among treatments. Beta diversity was assessed based on Bray–Curtis dissimilarity matrices and visualized using principal coordinates analysis (PCoA) and non-metric multidimensional scaling (NMDS). Differences in overall microbial community structure among treatments were tested using permutational multivariate analysis of variance (PERMANOVA) with 999 permutations.

Hierarchical clustering analysis was performed using the unweighted pair group method with arithmetic mean (UPGMA) based on Bray–Curtis dissimilarity. The goodness-of-fit between the dissimilarity matrix and the resulting dendrogram was assessed using the cophenetic correlation coefficient (r).

Core microbiome analysis was conducted to identify taxa consistently present across samples and within each dietary treatment group, based on prevalence thresholds. Correlation network analyses were performed using SECOM to evaluate treatment-dependent changes in microbial co-occurrence patterns, and divergence between networks was quantified using the MD-index.

Associations between bacterial taxa and dietary treatments were further explored using pattern search analysis based on Spearman rank correlation. Statistical analyses and ecological metrics were conducted using appropriate packages in R (vegan and related packages) and dedicated microbiome analysis tools, following standard ecological and microbiome analysis workflows.

## Results

### Performance and carcass yield

The effects of dietary treatments on the performance of European quail are presented in Table 3. Feed intake was not affected by the basal diet (*P* = 0.133), with similar values observed for birds fed corn–soybean meal and wheat–soybean meal diets (805.11 and 818.74 g/quail, respectively). However, quail fed wheat-based diets showed higher final body weight (298.15 vs. 282.92 g/quail; *P* = 0.002) and body weight gain (248.32 vs. 232.89 g/quail; *P* = 0.002), along with improved feed conversion ratio (3.32 vs. 3.46; *P* = 0.006), indicating better overall performance under this dietary condition.

**Table 3:** Feed intake, final body weight, body weight gain and feed conversion ratio of meat quail from 7 to 42-days-old fed to different basal diets, phytase and xylanase supplementation.

Basal diet	FI g/quail	FBW g/quail	BWG g/quail	FCR g/g
Corn + Soybean meal	805.11	282.92	232.89	3.46
Wheat + Soybean meal	818.74	298.15	248.32	3.32
P value	0.133	0.002	0.002	0.006
Phytase				
0	792.43	286.60	237.11	3.36
2000	831.43	294.48	244.10	3.42
P value	0.001	0.098	0.144	0.267
Xylanase				
0	805.77	292.28	242.55	3.34
16000	818.08	288.80	238.66	3.44
P value	0.174	0.460	0.411	0.056
Basal × Phytase	0.030	0.690	0.612	0.447
Basal × Xylanase	0.500	0.364	0.333	0.526
Phytase × Xylanase	0.026	0.009	0.010	0.078
Basal × Phytase × Xylanase	0.003	0.001	0.001	0.097
Average	811.926	290.538	240.604	3.389
SEM	6.314	3.299	3.321	0.035
C.V. (%)	4.12	6.01	7.3	5.49

FI = Feed intake (g/quail), FBW = final body weight (g/quail), BWG = body weight gain (g/quail), FCR = feed conversion ratio (FCR, g/g). SEM = standard error of the mean.

Phytase supplementation increased feed intake (831.43 vs. 792.43 g/quail; *P* = 0.001), without altering final body weight (*P* = 0.098), body weight gain (*P* = 0.144), or feed conversion ratio (*P* = 0.267), suggesting that the higher intake supported maintenance of performance. Similarly, xylanase supplementation did not significantly affect feed intake (*P* = 0.174), final body weight (*P* = 0.460), or body weight gain (*P* = 0.411), while feed conversion ratio showed a tendency (*P* = 0.056), indicating a subtle response depending on dietary context.

Significant interactions were observed for some variables. A basal diet × phytase interaction was detected for feed intake (*P* = 0.030), whereas other variables were not affected (*P* > 0.05). No significant basal diet × xylanase interactions were observed (*P* > 0.05). In contrast, phytase × xylanase interaction effects were significant for feed intake (*P* = 0.026), final body weight (*P* = 0.009), and body weight gain (*P* = 0.010), demonstrating that enzyme responses were interdependent. Additionally, significant three-way interactions (basal diet × phytase × xylanase) were observed for feed intake (*P* = 0.003), final body weight (*P* = 0.001), and body weight gain (*P* = 0.001), reinforcing that the effects of enzyme supplementation depend on the dietary matrix (Supplementary Tables 1–3). Feed conversion ratio was not influenced by interactions (*P* = 0.097).

Absolute and relative weights of carcass, edible viscera (heart, liver, and gizzard), and small intestine length of meat quail at 42 d of age are presented in Table 4. A significant effect of basal diet was observed for carcass yield (*P* = 0.013), with birds fed the corn–soybean meal diet showing higher values compared to those fed the wheat–soybean meal diet. Additionally, basal diet influenced liver and gizzard weights, both in relative terms to carcass and live body weight (*P* < 0.05), with corn-based diets increasing liver weight, whereas wheat-based diets resulted in greater gizzard weight.

**Table 4:** Carcass yield, relative weights of heart, liver, and gizzard in relation to carcass and live body weight, and small intestine length of European quails fed different basal diets with phytase or xylanase supplementation.

	SL	Carc	CRW. %			LRW. %		
Basal diet			Heart	Liver	Gizzard	Heart	Liver	Gizzard
Corn + Soybean meal	55.56	71.88^a^	1.91	4.83^a^	3.09^b^	1.37	3.45^a^	2.21^b^
Wheat + Soybean meal	54.39	70.11^b^	2.01	4.42^b^	3.69^a^	1.42	3.08^b^	2.59^a^
P value	0.161	0.013	0.304	0.049	0.001	0.527	0.007	0.001
Phytase								
0	54.19	70.20^b^	1.9	4.74	3.36	1.33	3.31	2.35
2000	55.76	71.79^a^	2.02	4.51	3.41	1.45	3.23	2.45
P value	0.751	0.025	0.207	0.261	0.707	0.100	0.538	0.308
Xylanase								
0	55.11	70.67	2.01	4.77	3.49	1.42	3.36	2.46
1600	54.84	71.33	1.91	4.47	3.29	1.37	3.18	2.34
P value	0.140	0.344	0.317	0.141	0.120	0.455	0.191	0.207
Basal × Phytase	0.205	0.909	0.506	0.387	0.168	0.522	0.339	0.137
Basal × Xylanase	0.667	0.096	0.097	0.415	0.168	0.185	0.602	0.322
Phytase × Xylanase	0.232	0.031	0.030	0.324	0.452	0.010	0.584	0.176
Basal × Phytase × Xylanase	0.353	0.168	0.234	0.398	0.042	0.137	0.183	0.015
Average	54.97	71.00	1.96	4.62	3.39	1.39	3.27	2.40
SEM	0.054	0.490	0.060	0.146	0.092	0.050	0.094	0.064
C.V. (%)	32.94	4.38	22.27	19.98	17.22	23.16	18.25	17.06

Carc = carcass yield (%); CRW = relative organ weight in relation to carcass (%); LRW = relative organ weight in relation to live body weight (%); SL = small intestine length (cm). SEM = standard error of the mean.

Phytase supplementation increased carcass yield (*P* = 0.025), with higher values observed at 2000 FTU, but did not affect the other evaluated parameters. No main effects of xylanase supplementation were detected for any variable (*P* > 0.05).

Regarding interactions, a significant phytase × xylanase interaction was observed for carcass yield (*P* = 0.031) and relative heart weight (LRW; *P* = 0.010) (Supplementary Tables 4, 5, and 7), indicating that the response to enzyme supplementation depended on their combined inclusion. A three-way interaction (basal diet × phytase × xylanase) was detected for gizzard weight (CRW; *P* = 0.042) and relative gizzard weight (LRW; *P* = 0.015), suggesting that the effects of enzyme supplementation varied according to the basal diet (Supplementary Tables 6 and 8). However, no significant effects were observed for small intestine length across treatments (*P* > 0.05).

### pH and myo-inositol

The effects of dietary treatments on digesta pH and inositol phosphate concentrations are presented in Table 5. Digesta pH was influenced by basal diet (6.31 vs. 6.14; *P* = 0.001) and phytase supplementation (6.32 vs. 6.14; *P* = 0.004), whereas xylanase had no effect (*P* = 0.147).

**Table 5:** pH and InsP concentration in the digesta of European quails fed different basal diets with supplementation of phytase or xylanase.

Basal diet	pH	InsP6	InsP5	InsP4	InsP3
Corn + Soybean meal	6.31^a^	6.62^a^	1.47^a^	0.87^a^	0.37^a^
Wheat + Soybean meal	6.14^b^	6.08^b^	1.06^b^	0.60^b^	0.55^b^
P value	0.001	0.001	0.001	0.001	0.001
Phytase					
0	6.32^a^	7.01^a^	1.44^a^	0.89^a^	0.58^a^
2000	6.14^b^	5.69^b^	1.09^b^	0.58^b^	0.34^b^
P value	0.004	0.001	0.001	0.001	0.001
Xylanase					
0	6.26	6.62^a^	1.43^a^	0.80	0.53^a^
1600	6.19	6.08^b^	1.10^b^	0.67	0.39^b^
P value	0.147	0.001	0.001	0.055	0.004
Basal × Phytase	0.244	0.001	0.830	0.695	0.023
Basal × Xylanase	0.244	0.095	0.454	0.109	0.919
Phytase × Xylanase	0.558	0.857	0.748	0.615	0.132
Basal × Phytase × Xylanase	0.558	0.095	0.028	0.015	0.760
Average	6.25	6.56	1.29	0.74	0.44
SEM	0.034	0.069	0.046	0.044	0.024
C.V. (%)	2.91	5.81	19.58	32.33	28.48

Basal diet significantly affected all inositol phosphate fractions, with higher concentrations observed in birds fed corn–soybean meal diets compared with wheat–soybean meal diets for InsP6 (6.62 vs. 6.08), InsP5 (1.47 vs. 1.06), InsP4 (0.87 vs. 0.60), and InsP3 (0.37 vs. 0.55; *P* = 0.001).

Phytase supplementation reduced the concentrations of all evaluated inositol phosphates, including InsP6 (7.01 vs. 5.69), InsP5 (1.44 vs. 1.09), InsP4 (0.89 vs. 0.58), and InsP3 (0.58 vs. 0.34; *P* ≤ 0.001). Similarly, xylanase supplementation reduced InsP6 (6.62 vs. 6.08; *P* = 0.001), InsP5 (1.43 vs. 1.10; *P* = 0.001), and InsP3 (0.53 vs. 0.39; *P* = 0.004), while no effect was observed for InsP4 (*P* = 0.055).

A significant basal diet × phytase interaction was observed for InsP6 (*P* = 0.001) and InsP3 (*P* = 0.023) (Supplementary Tables 9 and 12), whereas no interaction effects were detected for pH, InsP5, or InsP4 (*P* > 0.05). No significant basal diet × xylanase or phytase × xylanase interactions were observed for any variable (*P* > 0.05).

A significant three-way interaction (basal diet × phytase × xylanase) was detected for InsP5 (*P* = 0.028) and InsP4 (*P* = 0.015), while pH, InsP6, and InsP3 were not affected (*P* > 0.05) (Supplementary Tables 10 and 11).

### Gut microbiota

**Fig. 1:**
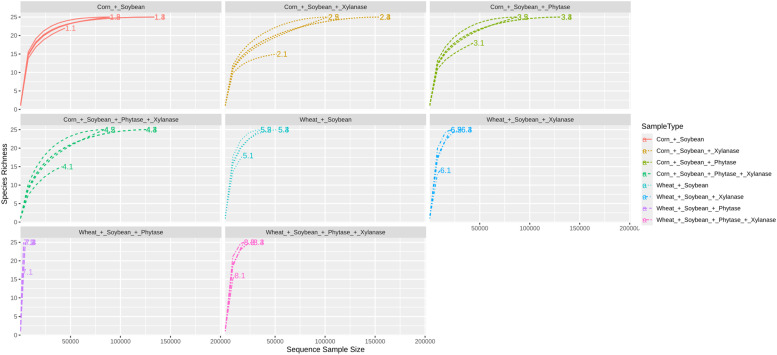
Rarefaction curves showing the observed species richness across samples. Each curve represents the cumulative number of observed operational taxonomic units (OTUs) as a function of sequencing depth. Curves approaching a plateau indicate that sequencing coverage was sufficient to capture most of the microbial diversity, while curves still rising suggest potential under-sampling.

Rarefaction analysis indicated sufficient sequencing depth across all samples, as curves approached saturation and showed comparable coverage among treatments (Fig. 1).

**Fig. 2:**
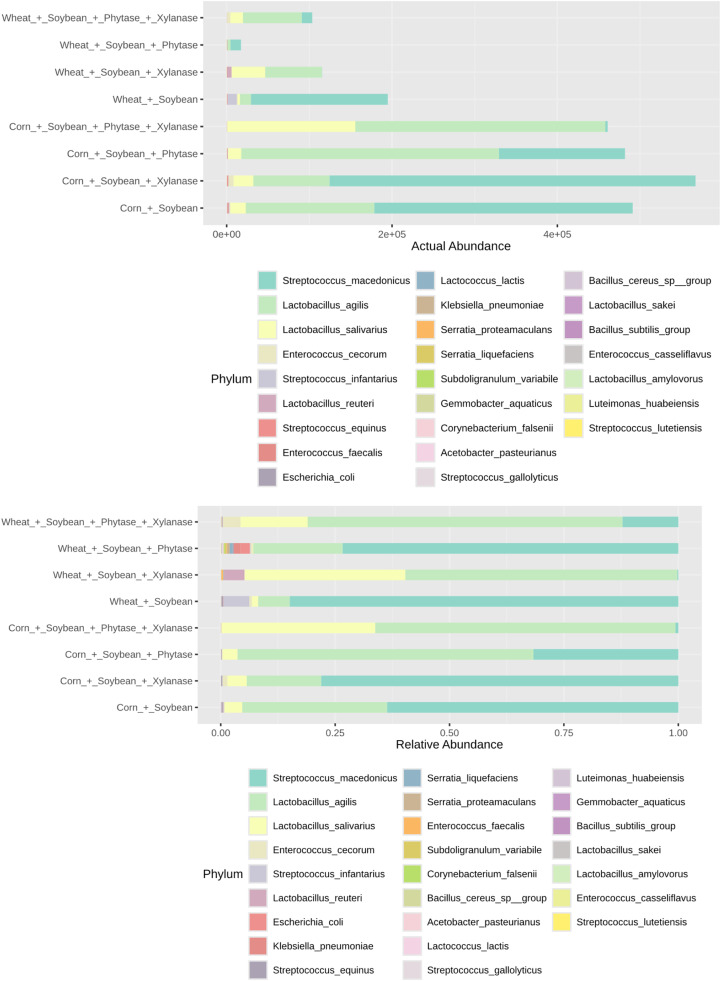
Abundance of taxa across samples. The upper panel shows absolute abundance (number of sequences per taxon), while the lower panel shows relative abundance (percentage of each taxon within the total community). This dual representation allows comparison of both overall quantity and proportional composition of the microbial communities.

Overall taxonomic composition and abundance patterns across samples are shown in Fig. 2, illustrating both absolute and relative abundances of bacterial taxa among dietary treatments.

**Fig. 3:**
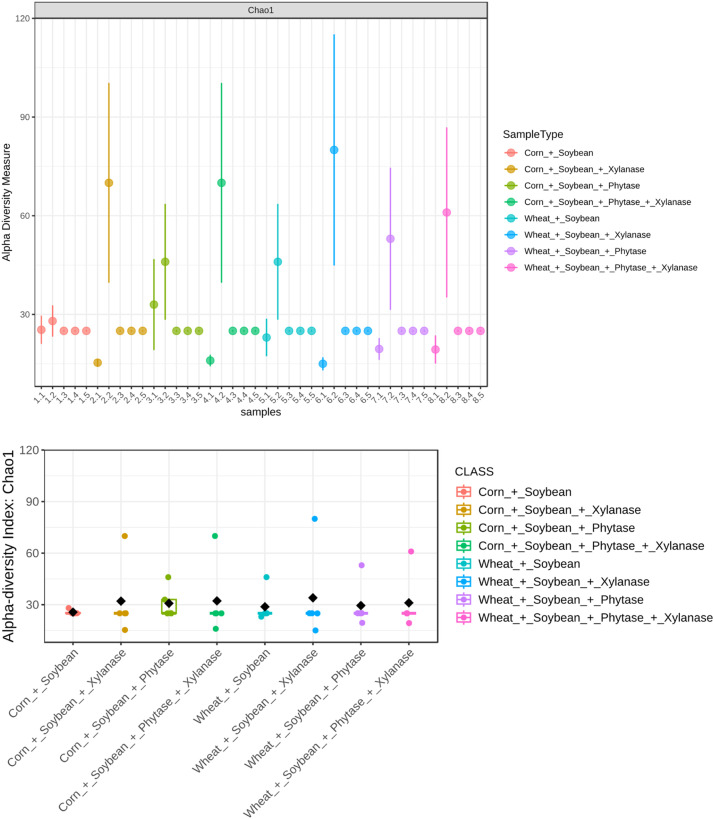
Alpha diversity of microbial communities assessed using the Chao1 richness index. Statistical comparisons among groups were performed using Welch’s t-test and one-way ANOVA. No significant differences were observed (p = 0.997; ANOVA F = 0.115), indicating comparable species richness across all samples.

**Fig. 4:**
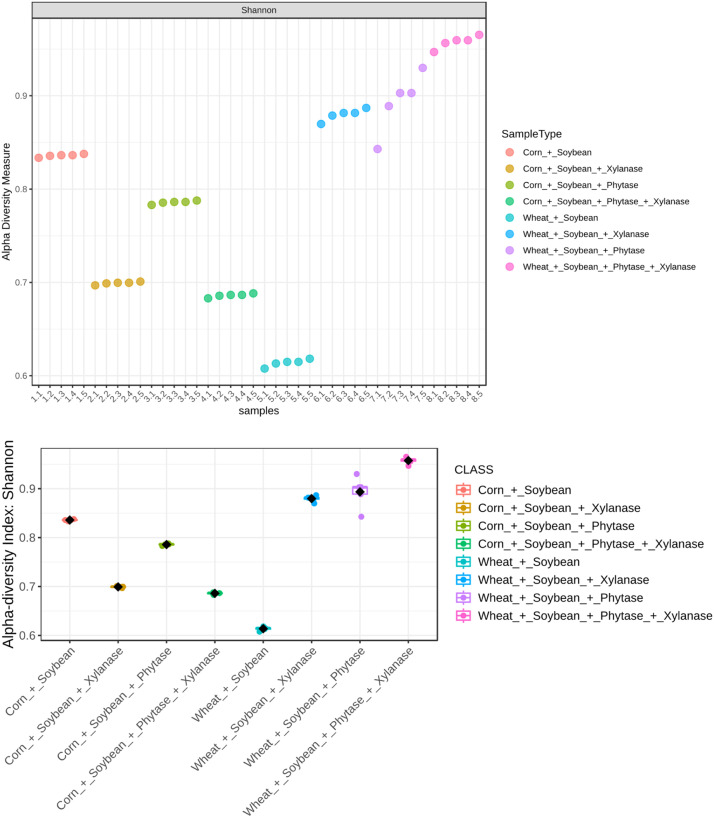
Alpha diversity of microbial communities assessed using the Shannon diversity index. Statistical comparisons among groups were performed using Welch’s t-test and one-way ANOVA. Significant differences were observed among groups (p = 8.10 × 10⁻³¹; ANOVA F = 499.79), indicating notable variation in both richness and evenness of the microbial communities across samples.

Alpha diversity analysis demonstrated that dietary treatments did not affect estimated bacterial richness, as reflected by the Chao1 index (Welch ANOVA: *F* = 0.115; *p* = 0.997; Fig. 3). In contrast, Shannon diversity differed markedly among treatments (Welch ANOVA: *F* = 499.79; *p* = 8.10 × 10⁻³¹; Fig. 4), indicating significant treatment effects on microbial community evenness.

**Fig. 5:**
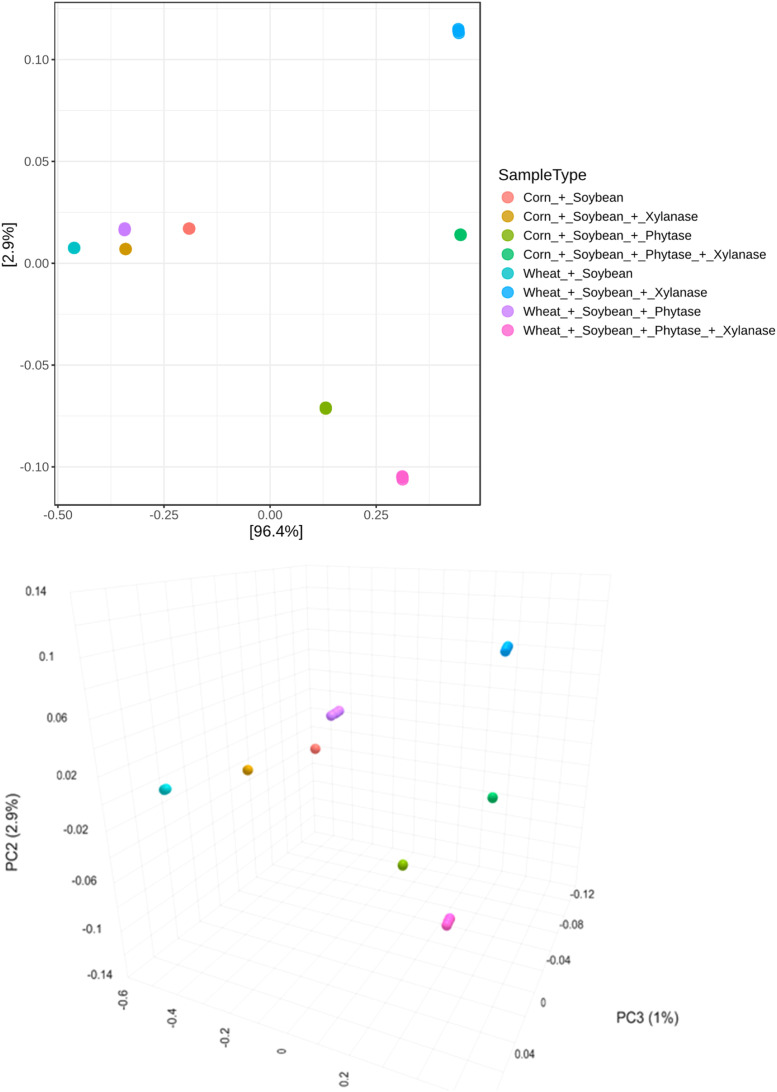
Beta diversity of microbial communities visualized using Principal Coordinates Analysis (PCoA). Statistical differences among groups were evaluated using PERMANOVA, revealing significant separation of communities (F = 226.62; R² = 0.322; P = 0.001), indicating strong differences in overall community composition between the groups.

**Fig. 6:**
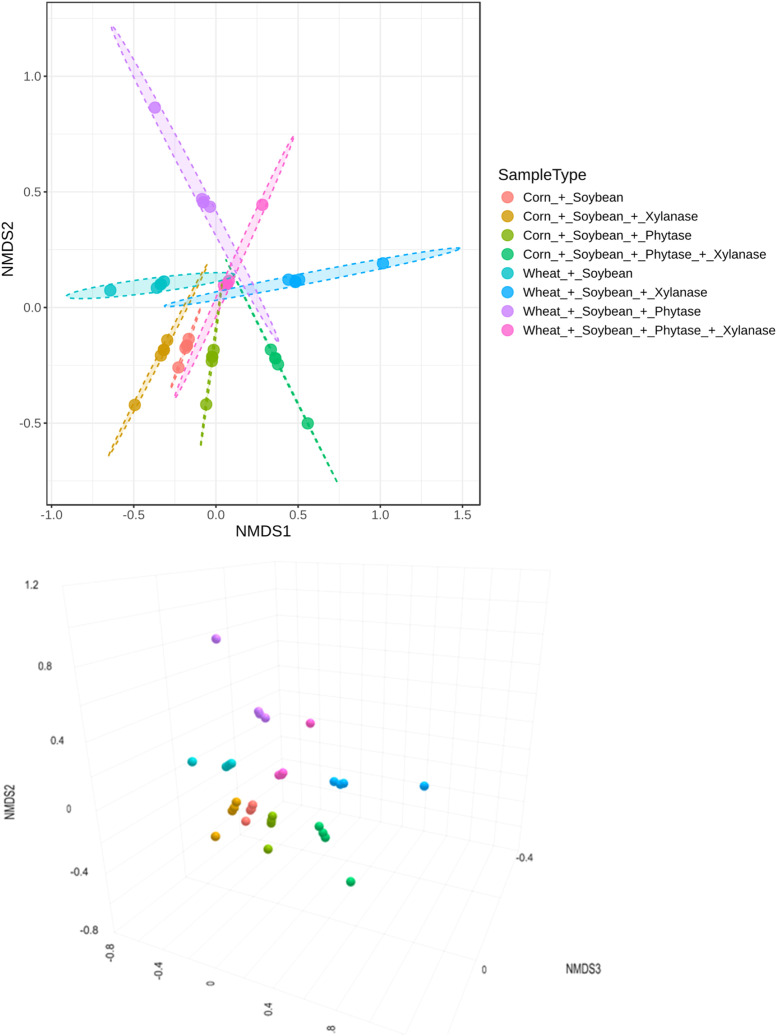
Beta diversity of microbial communities visualized using Non-metric Multidimensional Scaling (NMDS). Statistical differences among groups were assessed using PERMANOVA, showing significant separation of communities (F = 226,62; R² = 0.322; p = 0.001). The NMDS stress value of 0.109 indicates a good representation of the dissimilarities in two-dimensional space.

Beta diversity analysis based on Bray–Curtis dissimilarity revealed a clear separation of intestinal microbial communities according to dietary treatments (Fig. 5). PERMANOVA confirmed a highly significant treatment effect (*F* = 226,62; R² = 0.322; *p* = 0.001). These results were supported by NMDS ordination, which showed robust clustering with a low stress value (0.109; Fig. 6).

**Fig. 7:**
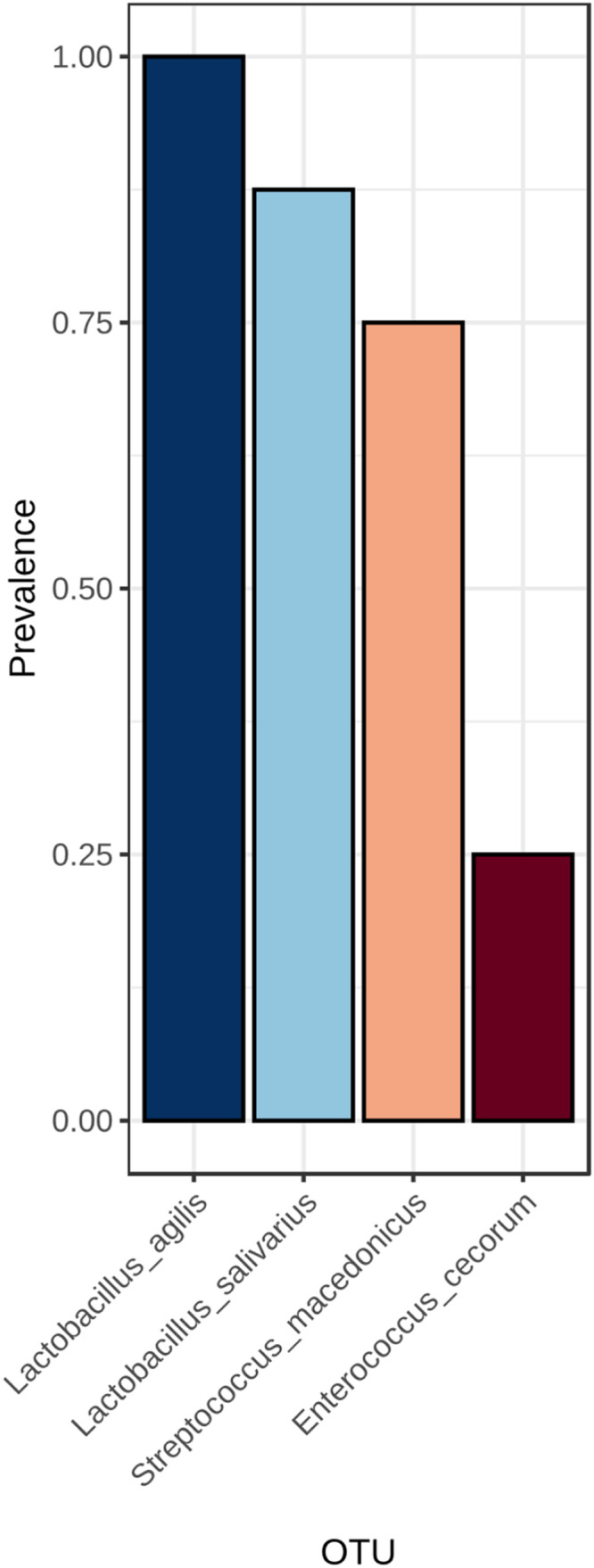
Core microbiome analysis across all samples. Taxa present in all samples are identified, highlighting the shared microbial community members that are consistently detected across the dataset. This analysis provides insight into the stable and prevalent microbial taxa within the studied population.

**Fig. 8:**
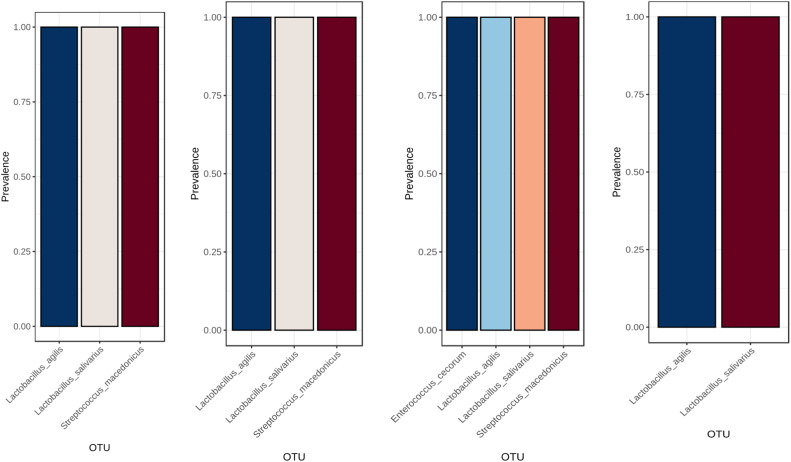
Core microbiome analysis of microbial communities across different dietary treatments: Corn + Soybean, Corn + Soybean + Phytase, Corn + Soybean + Xylanase, and Corn + Soybean + Phytase + Xylanase, respectively. Taxa present in all samples within each treatment are identified, highlighting the shared and consistently detected microbial members for each dietary group.

**Fig. 9:**
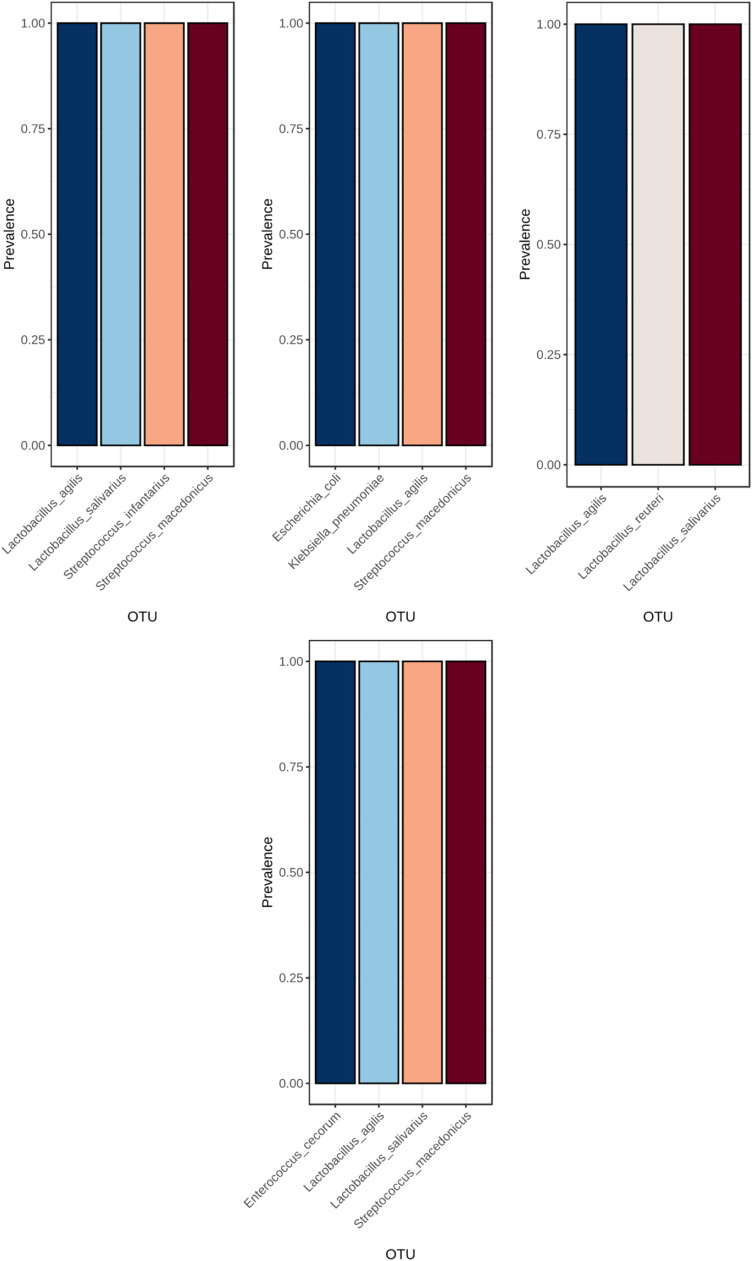
Core microbiome analysis of microbial communities across different dietary treatments: Wheat + Soybean, Wheat + Soybean + Phytase, Wheat + Soybean + Xylanase, and Wheat + Soybean + Phytase + Xylanase, respectively. Taxa present in all samples within each treatment are identified, highlighting the shared and consistently detected microbial members for each dietary group.

Core microbiome analysis identified a stable set of bacterial taxa consistently present across all samples, representing the intestinal core microbiota of meat quail (Fig. 7). Within corn–soybean-based diets, enzyme supplementation altered the prevalence of specific core taxa (Fig. 8). Similarly, wheat-soybean-based diets exhibited distinct enzyme-dependent core microbiome patterns (Fig. 9).

**Fig. 10:**
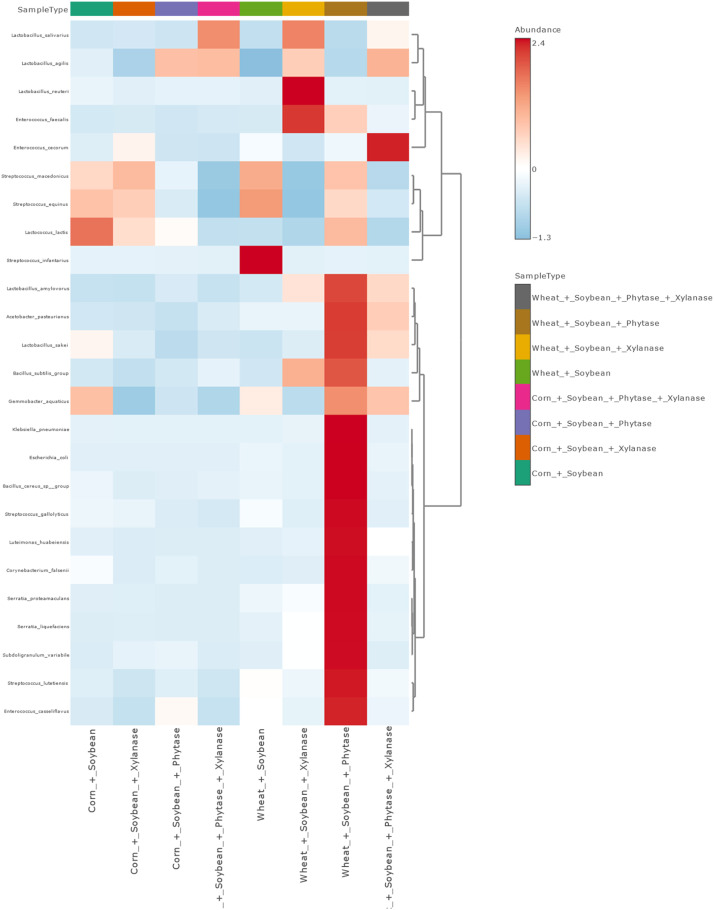
Clustering heatmap of microbial taxa abundance across samples. Rows represent taxa and columns represent samples, with colors indicating relative abundance levels. Hierarchical clustering was performed to group similar taxa and samples, highlighting patterns of microbial community composition and similarities among treatments.

Hierarchical clustering heatmap analysis revealed strong grouping of samples by dietary treatment (Fig. 10), corroborating beta diversity results.

**Fig. 11:**
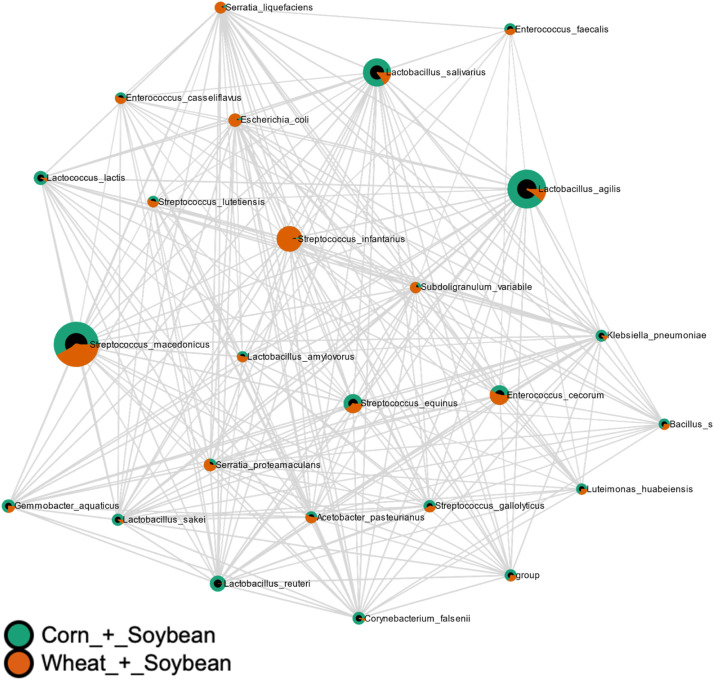
Correlation analysis of microbial communities using SECOM (Pearson correlation). Comparisons were made between Corn + Soybean and Wheat + Soybean treatments. The MD-index of −0.2855 indicates a negative correlation between the microbial profiles of the two dietary groups.

**Fig. 12:**
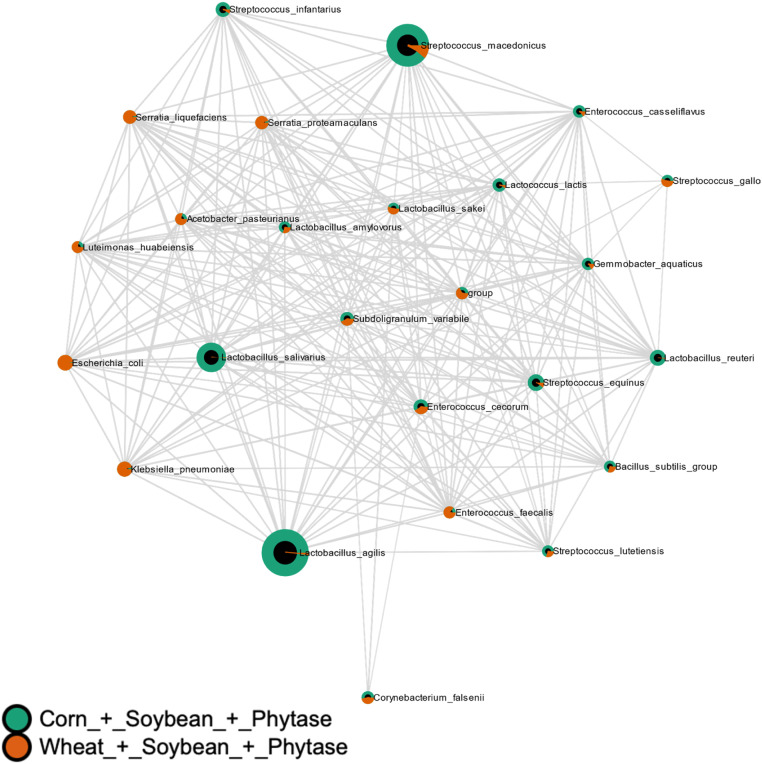
Correlation analysis of microbial communities using SECOM (Pearson correlation). Comparisons were made between Corn + Soybean + Phytase and Wheat + Soybean + Phytase treatments. The MD-index of −3.4578 indicates a strong negative correlation between the microbial profiles of the two dietary groups.

**Fig. 13:**
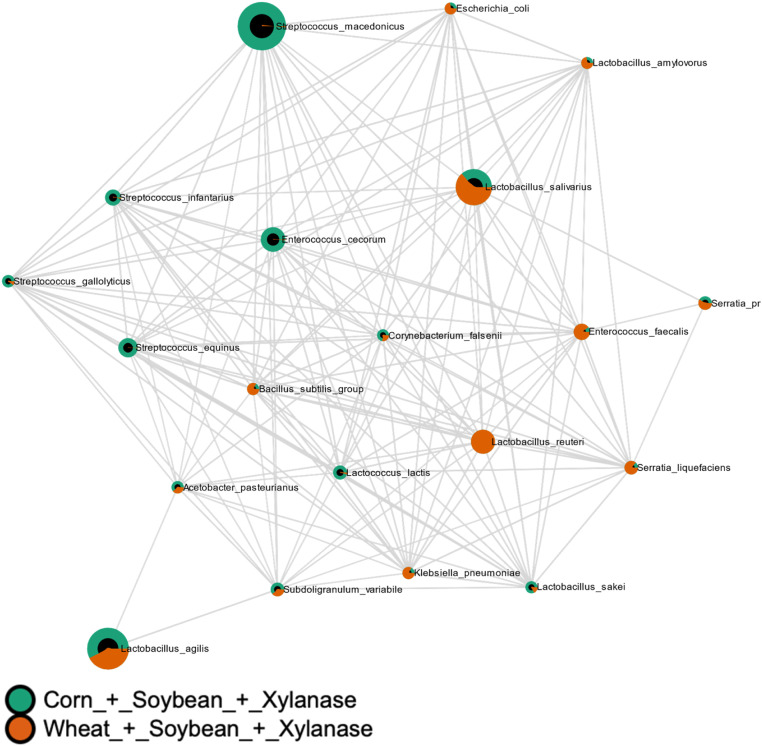
Correlation analysis of microbial communities using SECOM (Pearson correlation). Comparisons were made between Corn + Soybean + Xylanase and Wheat + Soybean + Xylanase treatments. The MD-index of 1.9349 indicates a positive correlation between the microbial profiles of the two dietary groups.

**Fig. 14:**
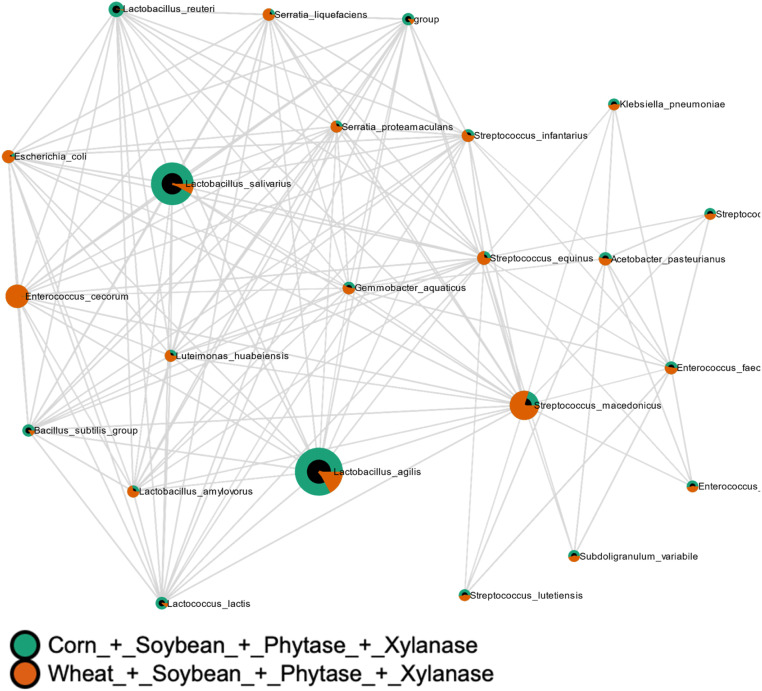
Correlation analysis of microbial communities using SECOM (Pearson correlation). Comparisons were made between Corn + Soybean + Phytase + Xylanase and Wheat + Soybean + Phytase + Xylanase treatments. The MD-index of −0.6253 indicates a moderate negative correlation between the microbial profiles of the two dietary groups.

Correlation network analysis showed treatment-dependent reorganization of microbial interaction patterns. Basal diet alone induced moderate divergence in microbial networks (MD-index = −0.2855; Fig. 11). Phytase supplementation resulted in pronounced matrix-dependent divergence (MD-index = −3.4578; Fig. 12), whereas xylanase supplementation also induced strong, diet-dependent reorganization (MD-index = 1.9349; Fig. 13). Combined supplementation of phytase and xylanase produced moderate divergence (MD-index = −0.6253; Fig. 14).

**Fig. 15:**
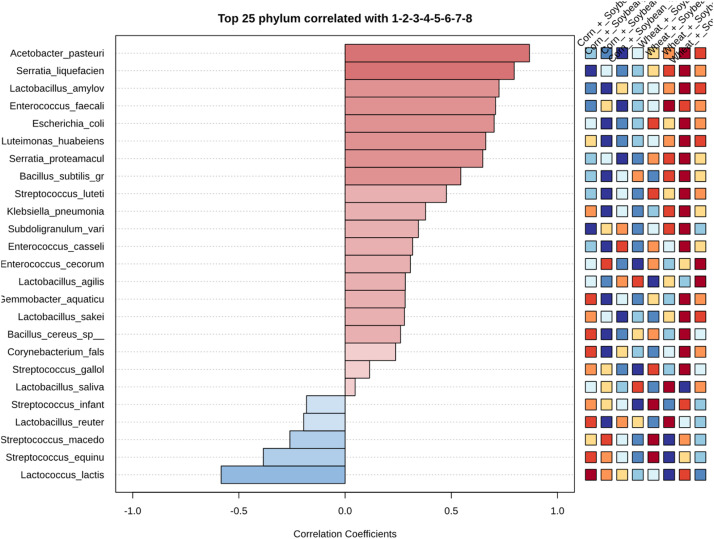
Pattern search analysis of microbial communities using Spearman rank correlation as the distance measure. This analysis identifies consistent patterns of co-occurrence and relationships among taxa across samples, highlighting correlations in microbial profiles within and between treatments.

Pattern search analysis identified bacterial taxa whose abundance patterns were significantly associated with specific dietary treatments based on Spearman rank correlation (Fig. 15).

## Discussion

The present study demonstrates that basal diet composition and enzyme supplementation strategy interact to modulate productive performance, carcass traits, and intestinal microbial ecology in meat-type quail. Collectively, the results indicate that wheat–soybean meal-based diets supported improved growth performance compared with corn-based diets, while enzyme supplementation modulated performance responses in a matrix-dependent manner and induced marked restructuring of the intestinal microbiota. These findings highlight the central role of feed matrix characteristics in determining both productive and microbial responses to exogenous enzymes.

Importantly, these results address a critical knowledge gap regarding the combined effects of phytase and xylanase in quail. While both enzymes are widely used in poultry nutrition, their interactive effects on nutrient utilization and intestinal microbial ecology in quail have remained poorly characterized. The present study provides evidence that enzyme interactions in wheat-based diets extend beyond digestibility-related responses and are associated with coordinated modulation of the intestinal microbial ecosystem, as reflected by changes in inositol phosphate degradation patterns and microbial diversity indices.

The performance differences observed between basal diets may be associated with the distinct physicochemical characteristics of feed ingredients. Wheat-based diets resulted in higher body weight gain and improved feed efficiency, which may reflect differences in nutrient accessibility and digestive dynamics. Although enzyme supplementation did not consistently improve performance parameters, the observed interaction effects indicate that responses to phytase and xylanase depend on dietary context, particularly in wheat-based formulations where structural constraints may limit nutrient availability.

In wheat, most phytate is localized in the aleurone layer, with a substantial fraction embedded within thick, arabinoxylan-rich cell walls (O’Dell et al., 1972; Pekkinen et al., 2014). This structural complexity limits phytase access to phytate ( Paloheimo et al., 2010). Xylanase-mediated hydrolysis of arabinoxylans can disrupt these barriers, increasing phytate accessibility and potentially enhancing phytase efficacy. This mechanism is consistent with previous reports demonstrating improved phytate degradation and nutrient utilization when phytase and xylanase are used together in wheat-based diets (Selle et al., 2009; Zeller et al., 2015) and supports the interaction effects observed in the present study.

The reduction in inositol phosphate concentrations following phytase supplementation confirms the effective hydrolysis of phytate, as evidenced by lower levels of InsP6, InsP5, InsP4, and InsP3 in the digesta. These results are consistent with the established mode of action of phytase and indicate increased release of phosphorus and other bound nutrients. Xylanase supplementation also reduced specific inositol phosphate fractions, suggesting that cell wall degradation may have facilitated greater substrate accessibility for endogenous or exogenous phytase activity.

Beyond improving phytate degradation, xylanase generates xylo-oligosaccharides (XOS) with recognized prebiotic properties (Aachary and Prapulla, 2011; Morgan et al., 2017). These compounds selectively stimulate saccharolytic bacteria and promote short-chain fatty acid production (Samanta et al., 2015), mechanisms that have been associated with improved gut function in poultry (Maesschalck et al., 2015). Although short-chain fatty acids were not measured, the observed restructuring of microbial communities and interaction networks is consistent with shifts toward increased fermentative activity.

In addition to the prebiotic effects of xylo-oligosaccharides, phytate hydrolysis by phytase generates lower-order inositol phosphates and free myo-inositol, which may exert modulatory effects on the intestinal microbial ecosystem (Jessen and Bui, 2025). The progressive dephosphorylation of phytate reduces its chelating capacity, increasing the availability of phosphorus and other minerals essential for both host metabolism and microbial growth (Ribeiro et al., 2024). Phosphorus availability is a key factor influencing microbial proliferation and metabolic activity (Li et al., 2022). Furthermore, intermediate degradation products (InsP5–InsP3) and myo-inositol (Naithani et al., 2026; Ribeiro et al., 2024) may serve as substrates for specific microbial groups, supporting metabolically specialized taxa and contributing to shifts in microbial composition and activity. These mechanisms are consistent with the changes observed in microbial community structure in the present study.

In this context, phytase supplementation may contribute not only to nutrient release but also to the modulation of the intestinal ecosystem by alleviating mineral limitations and influencing microbial resource availability (Borda-Molina et al., 2016). When combined with xylanase, which increases the supply of fermentable carbohydrates, a complementary effect may occur in which both carbon and phosphorus availability are enhanced. This combined nutrient release may help explain the interaction effects observed for microbial community structure and network organization.

The higher relative gizzard weight observed in birds fed wheat–soybean meal diets is consistent with the greater fiber content of these formulations. Increased dietary fiber enhances gizzard development and prolongs digesta retention time, improving mechanical and enzymatic digestion (González-Alvarado et al., 2007). This physiological adaptation may contribute to improved digestive efficiency and reinforce diet-dependent responses to enzyme supplementation.

Microbiota analyses demonstrated that dietary treatments did not affect estimated bacterial richness, as reflected by the Chao1 index (*P* = 0.99685). In contrast, Shannon diversity differed markedly among treatments (*p* = 8.10 × 10⁻³¹), indicating that dietary treatments primarily influenced microbial community evenness and dominance structure rather than total taxonomic richness. This pattern is consistent with previous reports showing that phytase and xylanase modulate microbial community structure through changes in substrate availability and gut environment (Hoeck et al., 2021; Moita, 2023).

This selective restructuring of community evenness aligns with previous findings that exogenous enzymes modify the intestinal ecological environment. Xylanase supplementation has been shown to induce shifts in the cecal microbiome, increasing saccharolytic taxa such as Enterococcus and Lactobacillales (Hoeck et al., 2021). Similarly, phytase supplementation has been associated with reductions in potentially harmful bacteria and improvements in intestinal health (Moita, 2023). These findings support the interpretation that enzyme supplementation primarily alters microbial structure and dominance patterns rather than overall richness.

Beta diversity analyses revealed a clear separation of microbial communities according to dietary treatments, confirming that both basal diet and enzyme supplementation are major drivers of microbial structure. This matrix-dependent effect is consistent with previous studies demonstrating that enzyme efficacy is strongly influenced by diet composition, particularly fiber and phytate content (Anadón et al., 2019; Kiarie et al., 2013).

Core microbiome analysis identified a stable set of taxa across treatments, although their relative abundance varied depending on diet and enzyme supplementation. Wheat-based diets exhibited greater responsiveness to enzyme-induced shifts, supporting the concept that xylanase exerts stronger microbiota-modulating effects in diets rich in non-starch polysaccharides (Kiarie et al., 2013; Liu et al., 2023).

Correlation network analyses further demonstrated that enzyme supplementation altered microbial interaction patterns in a matrix-dependent manner. Phytase supplementation resulted in pronounced network divergence, potentially due to changes in phosphorus availability and myo-inositol release, which may act as ecological drivers of microbial interactions rather than classical prebiotic substrates (Hoeck et al., 2021; Liu et al., 2023; Moita, 2023; Widodo et al., 2018). Xylanase supplementation also induced strong network reorganization, consistent with increased availability of fermentable substrates and stimulation of saccharolytic microbial pathways (Hoeck et al., 2021; Widodo et al., 2018).

Interestingly, combined enzyme supplementation resulted in moderate network divergence, suggesting interactive effects that may promote a more balanced microbial ecosystem. This pattern supports previous observations that combined enzyme strategies can modulate gut ecology through complementary mechanisms (Anadón et al., 2019; Liu et al., 2023).

Pattern search analysis reinforced these findings by identifying taxa associated with specific dietary treatments. Overall, the results demonstrate that enzyme supplementation acts not only on nutrient availability but also as a modulator of microbial community structure, with effects strongly dependent on basal diet composition (Kiarie et al., 2013; Liu et al., 2023; Tajudeen et al., 2025).

Although the present study provides robust evidence of enzyme-driven microbial modulation, functional outputs such as short-chain fatty acid concentrations and myo-inositol levels were not directly measured. Future studies integrating metabolomic approaches will be important to establish direct links between microbial restructuring and host physiological responses.

Overall, wheat–soybean meal-based diets supported improved performance, while enzyme supplementation modulated both productive responses and intestinal microbial ecology in a diet-dependent manner. These findings demonstrate that phytase and xylanase act not only as digestibility enhancers but also as important modulators of gut microbial structure, with effects strongly influenced by the dietary matrix.

## Conclusion

Phytase and xylanase supplementation modulated gut microbial community structure in a basal diet-dependent manner in meat-type quail. Productive performance responses were influenced by the interaction between enzyme supplementation and diet composition, rather than by consistent main effects of enzymes. Basal diet characteristics and enzyme strategy were key determinants of microbial organization, highlighting the importance of enzyme - matrix interactions in shaping nutrient utilization and intestinal microbial ecology, as well as the need to consider diet composition when evaluating enzyme efficacy in poultry systems.

## Disclosures

The authors declare that they have no known competing financial or personal interest that could have impact the outcome of the study reported in this paper.

## CRediT authorship contribution statement

**Iva Carla de Barros Ayres:** Writing – review & editing, Writing – original draft, Methodology, Investigation, Formal analysis, Data curation, Conceptualization. **Adiel Vieira de Lima:** Writing – review & editing, Formal analysis, Data curation, Conceptualization. **Aline Beatriz Rodrigues:** Formal analysis, Data curation, Conceptualization. **Paloma Eduarda Lopes de Souza:** Formal analysis, Data curation, Conceptualization. **Carlos Henrique do Nascimento:** Investigation, Data curation, Conceptualization. **Marcos Aurelio Victor de Assunção:** Investigation, Formal analysis, Data curation, Conceptualization. **Danilo Vargas Gonçalves Vieira:** Methodology, Formal analysis, Data curation, Conceptualization. **Alexandre Barbosa de Brito:** Formal analysis, Data curation, Conceptualization. **Apolônio Gomes Ribeiro:** Writing – review & editing, Formal analysis, Data curation, Conceptualization. **Ricardo Romão Guerra:** Writing – review & editing, Formal analysis, Data curation, Conceptualization. **Thácyla Beatriz Duarte Correia:** Formal analysis, Data curation, Conceptualization. **José de Arimatéia de Freitas Pinto:** Formal analysis, Data curation, Conceptualization. **Fernando Guilherme Perazzo Costa:** Writing – review & editing, Writing – original draft, Project administration, Methodology, Funding acquisition, Formal analysis, Data curation, Conceptualization. **Lucas Rannier Ribeiro Antonino Carvalho:** Writing – review & editing, Formal analysis, Data curation, Conceptualization. **Matheus Ramalho de Lima:** Writing – review & editing, Writing – original draft, Supervision, Resources, Project administration, Methodology, Investigation, Funding acquisition, Formal analysis, Data curation, Conceptualization.

## Appendix Supplementary materials

Image, application 1

## Footnotes

Supplementary material associated with this article can be found, in the online version, at doi:10.1016/j.psj.2026.106935.
