# Supplementary material for: Xylanase and phytase as modulators of gut microbiota and phytate degradation in wheat-based diets for meat quail
Source: Poult Sci. 2026 Apr 15;105(7):106935. doi: 10.1016/j.psj.2026.106935 (PMC13125166; doi:10.1016/j.psj.2026.106935)
Supplement: Supplementary file 1 [file mmc1.docx]

**Supplementary Tables**

**Supplementary Table 1.** Unfolding of the significant interaction between basal diet, phytase, and xylanase supplementation for feed intake (FI, g/quail) of meat quail from 7 to 42 days of age.

| Phytase | Corn + Soybean meal | Wheat + Soybean meal | *P-value* |
| --- | --- | --- | --- |
| 0 | 795.6 | 789.3 B | 0.619 |
| 2000 | 814.6 b | 848.2 Aa | 0.011 |
| *P-value* | 0.138 | <0.001 |  |
| SEM | 8.930 | |  |
| Phytase | Xylanase | | *P-value* |
|  | 0 | 16000 |  |
| 0 | 796.5 | 788.3 B | 0.520 |
| 2000 | 815.0 b | 847.8 Aa | 0.012 |
| *P-value* | 0.149 | <0.001 |  |
| SEM | 8.930 | |  |
| Phytase - Xylanase | Corn + Soybean meal | Wheat + Soybean meal | *P-value* |
| 0 Phytase; 0 Xylanase | 779.5 b | 813.6 a | 0.062 |
| 0 Phytase; 16000 Xylanase | 811.7 a | 764.9 b | 0.012 |
| 2000 Phytase; 0 Xylanase | 812.4 | 817.7 | 0.769 |
| 2000 Phytase; 16000 Xylanase | 816.9 b | 878.8 a | 0.001 |
| SEM | 12.628 | |  |
| Basal - Xylanase | Phytase | | *P-value* |
|  | 0 | 2000 |  |
| Corn - 0 Xylanase | 779.5 b | 812.4 a | 0.071 |
| Corn - 16000 Xylanase | 811.7 | 816.9 | 0.775 |
| Wheat - 0 Xylanase | 813.6 | 817.7 | 0.820 |
| Wheat - 16000 Xylanase | 764.9 b | 878.8 a | <0.001 |
| SEM | 12.628 | |  |
| Basal - Phytase | Xylanase | | *P-value* |
|  | 0 | 16000 |  |
| Corn - 0 Phytase | 779.5 b | 811.7 a | 0.077 |
| Corn - 2000 Phytase | 812.4 | 816.9 | 0.804 |
| Wheat - 0 Phytase | 813.6 a | 764.9 b | 0.009 |
| Wheat - 2000 Phytase | 817.7 b | 878.8 a | 0.001 |
| SEM | 12.628 | |  |

Means followed by different uppercase letters within rows and lowercase letters within columns differ (P < 0.05). SEM = standard error of the mean.

**Supplementary Table 2.** Unfolding of the significant interaction between basal diet, phytase, and xylanase supplementation for final body weight (FBW, g/quail) of meat quail from 7 to 42 days of age.

| Phytase | Xylanase | | *P-value* |
| --- | --- | --- | --- |
|  | 0 | 16000 |  |
| 0 | 294.7 a | 278.5 Bb | 0.018 |
| 2000 | 289.9 | 299.1 A | 0.169 |
| *P-value* | 0.469 | 0,003 |  |
| SEM | 4.666 | |  |
| Phytase - Xilanase | Corn + Soybean meal | Wheat + Soybean meal | *P-value* |
| 0 Phytase; 0 Xylanase | 277.5 b | 311.9 a | 0.001 |
| 0 Phytase; 16000 Xylanase | 282.3 | 274.7 | 0.417 |
| 2000 Phytase; 0 Xylanase | 287.5 | 292.2 | 0.619 |
| 2000 Phytase; 16000 Xylanase | 284.3 b | 313.9 a | 0.003 |
| SEM | 6.598 | |  |
| Basal - Xylanase | Phytase | | *P-value* |
|  | 0 | 2000 |  |
| Corn - 0 Xylanase | 277.5 | 287.5 | 0.288 |
| Corn - 16000 Xylanase | 282.3 | 284.3 | 0.832 |
| Wheat - 0 Xylanase | 311.9 a | 292.2 b | 0.040 |
| Wheat - 16000 Xylanase | 274.7 b | 313.9 a | 0.001 |
| SEM | 6.598 | |  |
| Basal - Phytase | Xylanase | | *P-value* |
|  | 0 | 16000 |  |
| Corn – 0 Phytase | 277.5 | 282.3 | 0.607 |
| Corn - 2000 Phytase | 287.5 | 284.3 | 0.732 |
| Wheat - 0 Phytase | 311.9 a | 274.7 b | 0.002 |
| Wheat - 2000 Phytase | 292.2 b | 313.9 a | 0.025 |
| SEM | 6.598 | |  |

Means followed by different uppercase letters within rows and lowercase letters within columns differ (P < 0.05). SEM = standard error of the mean.

**Supplementary Table 3.** Unfolding of the significant interaction between basal diet, phytase, and xylanase supplementation for body weight gain (BWG, g/quail) of meat quail from 7 to 42 days of age.

| Phytase | Xylanase | | *P-value* |
| --- | --- | --- | --- |
|  | 0 | 16000 |  |
| 0 | 245.3 | 228.9 | 0.017 |
| 2000 | 239.8 | 248.4 | 0.200 |
| *P-value* | 0.408 | 0.005 |  |
| SEM | 4.696 | |  |
| Phytase - Xylanase | Corn + Soybean meal | Wheat + Soybean meal | *P-value* |
| 0 Phytase; 0 Xylanase | 228.2 | 262.5 | 0.006 |
| 0 Phytase; 16000 Xylanase | 233.0 | 224.8 | 0.387 |
| 2000 Phytase; 0 Xylanase | 236.9 | 242.7 | 0.542 |
| 2000 Phytase; 16000 Xylanase | 233.5 | 263.4 | 0.003 |
| SEM | 6.642 | |  |
| Basal - Xylanase | Phytase | | *P-value* |
|  | 0 | 2000 |  |
| Corn - 0 Xylanase | 228.2 | 236.9 | 0.358 |
| Corn - 16000 Xylanase | 233.0 | 233.5 | 0.962 |
| Wheat - 0 Xylanase | 262.5 | 242.7 | 0.040 |
| Wheat - 16000 Xylanase | 224.8 | 263.4 | 0.002 |
| SEM | 6.642 | |  |
| Base - Fitase | Xylanase | | *P-value* |
|  | 0 | 16000 |  |
| Corn - 0 Phytase | 228.2 | 233.0 | 0.610 |
| Corn - 2000 Phytase | 236.9 | 233.5 | 0.717 |
| Wheat - 0 Phytase | 262.5 | 224.8 | 0.002 |
| Wheat - 2000 Phytase | 242.7 | 263.4 | 0.032 |
| SEM | 6.642 | |  |

Means followed by different uppercase letters within rows and lowercase letters within columns differ (P < 0.05). SEM = standard error of the mean.

**Supplementary Table 4.** Unfolding of the significant interaction between basal diet, phytase, and xylanase supplementation for carcass yield (Carc, %) of European quails.

| Phytase | Xylanase | | *P-value* |
| --- | --- | --- | --- |
|  | 0 | 16000 |  |
| 0 | 69.10 Bb | 71.30 A | 0.029 |
| 2000 | 72.23 a | 71.36 | 0.380 |
| *P-value* | 0.002 | 0.953 |  |
| SEM | 0.695 | |  |

Means followed by different uppercase letters within rows and lowercase letters within columns differ (P < 0.05). SEM = standard error of the mean.

**Supplementary Table 5.** Unfolding of the significant interaction between basal diet, phytase, and xylanase supplementation for relative heart weight to carcass (CRW, %) of European quails.

| Phytase | Xylanase | |  |
| --- | --- | --- | --- |
|  | 0 | 16000 | *P-value* |
| 0 | 1.84 b | 1.96 | 0.394 |
| 2000 | 2.18 Aa | 1.87 B | 0.026 |
| *P-value* | 0.016 | 0.505 |  |
| SEM | 0.097 | |  |

Means followed by different uppercase letters within rows and lowercase letters within columns differ (P < 0.05). SEM = standard error of the mean.

**Supplementary Table 6.** Unfolding of the significant interaction between basal diet, phytase, and xylanase supplementation for relative gizzard weight to carcass (CRW, %) of European quails.

| Phytase - Xylanase | Basal | | *P-value* |
| --- | --- | --- | --- |
|  | Corn | Wheat |  |
| 0 Phytase; 0 Xylanase | 3.25 | 3.58 | 0.208 |
| 0 Phytase; 16000 Xylanase | 3.06 | 3.57 | 0.055 |
| 2000 Phytase; 0 Xylanase | 2.95A | 4.18B | 0.001 |
| 2000 Phytase; 16000 Xylanase | 3.10 | 3.43 | 0.207 |
| SEM | 0.092 | |  |
| Basal - Xylanase | Phytase | | *P-value* |
|  | 0 | 2000 |  |
| Corn; 0 Xylanase | 3.25 | 2.95 | 0.248 |
| Corn; 16000 Xylanase | 3.06 | 3.10 | 0.883 |
| Wheat; 0 Xylanase | 3.58B | 4.18A | 0.025 |
| Wheat; 16000 Xylanase | 3.57 | 3.43 | 0.599 |
| SEM | 0.092 | |  |
| Basal - Phytase | Xylanase | | *P-value* |
|  | 0 | 16000 |  |
| Corn; 0 Phytase | 3.25 | 3.06 | 0.458 |
| Corn; 2000 Phytase | 2.95 | 3.10 | 0.573 |
| Wheat; 0 Phytase | 3.58 | 3.57 | 0.945 |
| Wheat; 2000 Phytase | 4.18A | 3.43B | 0.005 |
| SEM | 0.092 | |  |

Means followed by different uppercase letters within rows and lowercase letters within columns differ (P < 0.05). SEM = standard error of the mean.

**Supplementary Table 7.** Unfolding of the significant interaction between basal diet, phytase, and xylanase supplementation for relative heart weight to live body weight (LRW, %) of European quails.

| Phytase | Xylanase | | *P-value* |
| --- | --- | --- | --- |
|  | 0 | 16000 |  |
| 0 | 1.26 b | 1.40 | 0.181 |
| 2000 | 1.58 Aa | 1.33 B | 0.019 |
| *P-value* | 0.003 | 0.486 |  |
| SEM | 0.072 | |  |

Means followed by different uppercase letters within rows and lowercase letters within columns differ (P < 0.05). SEM = standard error of the mean.

**Supplementary Table 8.** Unfolding of the significant interaction between basal diet, phytase, and xylanase supplementation for relative gizzard weight to live body weight (LRW, %) of European quails.

| Phytase - Xylanase | Basal | | *P-value* |
| --- | --- | --- | --- |
|  | Corn | Wheat |  |
| 0 Phytase; 0 Xylanase | 2.30 | 2.40 | 0.596 |
| 0 Phytase; 16000 Xylanase | 2.17 B | 2.54 A | 0.046 |
| 2000 Phytase; 0 Xylanase | 2.15 B | 2.98 A | 0.001 |
| 2000 Phytase; 16000 Xylanase | 2.23 | 2.42 | 0.303 |
| SEM | 0.129 | |  |
| Basal - Xylanase | Phytase | | *P-value* |
|  | 0 | 2000 |  |
| Corn; 0 Xylanase | 2.30 | 2.15 | 0.424 |
| Corn; 16000 Xylanase | 2.17 | 2.23 | 0.743 |
| Wheat; 0 Xylanase | 2.40 B | 2.98 A | 0.002 |
| Wheat; 16000 Xylanase | 2.54 | 2.42 | 0.507 |
| SEM | 0.129 | |  |
| Basal - Phytase | Xylanase | | *P-value* |
|  | 0 | 16000 |  |
| Corn; 0 Phytase | 2.30 | 2.17 | 0.48 |
| Corn; 2000 Phytase | 2.15 | 2.23 | 0.669 |
| Wheat; 0 Phytase | 2.40 | 2.54 | 0.428 |
| Wheat; 2000 Phytase | 2.98 A | 2.42 B | 0.003 |
| SEM | 0.129 | |  |

Means followed by different uppercase letters within rows and lowercase letters within columns differ (P < 0.05). SEM = standard error of the mean.

**Supplementary Table 9.** Unfolding of the significant interaction between basal diet, phytase, and xylanase supplementation for inositol hexakisphosphate (InsP6) concentration in the digesta of European quails.

| Phytase | Basal | | *P-value* |
| --- | --- | --- | --- |
|  | Corn | Wheat |  |
| 0 | 6.99 a | 7.03 a | 0.815 |
| 2000 | 6.25 Ab | 5.14 Bb | 0.001 |
| *P-value* | 0.001 | 0.001 |  |
| SEM | 0.098 | |  |

Means followed by different uppercase letters within rows and lowercase letters within columns differ (P < 0.05). SEM = standard error of the mean.

**Supplementary Table 10.** Unfolding of the significant interaction between basal diet, phytase, and xylanase supplementation for inositol pentakisphosphate (InsP5) concentration in the digesta of European quails.

| Phytase - Xylanase | Basal | | *P-value* |
| --- | --- | --- | --- |
|  | Corn | Wheat |  |
| 0 Phytase; 0 Xylanase | 1.70 | 1.50 | 0.137 |
| 0 Phytase; 16000 Xylanase | 1.59 A | 0.99 B | 0.001 |
| 2000 Phytase; 0 Xylanase | 1.53 A | 1.00 B | 0.002 |
| 2000 Phytase; 16000 Xylanase | 1.07 A | 0.74 B | 0.017 |
| SEM | 0.093 | |  |
| Basal - Xylanase | Phytase | | *P-value* |
|  | 0 | 2000 |  |
| Corn; 0 Xylanase | 1.70 | 1.53 | 0.201 |
| Corn; 16000 Xylanase | 1.59 A | 1.07 B | 0.003 |
| Wheat; 0 Xylanase | 1.50 A | 1.00 B | 0.004 |
| Wheat; 16000 Xylanase | 0.99 | 0.74 | 0.073 |
| SEM | 0.093 | |  |
| Basal - Phytase | Xylanase | | *P-value* |
|  | 0 | 16000 |  |
| Corn; 0 FIT | 1.70 | 1.59 | 0.3922 |
| Corn; 2000 FIT | 1.53 A | 1.07 B | 0.0012 |
| Wheat; 0 FIT | 1.50 A | 0.99 B | 0.0003 |
| Wheat; 2000 FIT | 1.00 | 0.74 | 0.0579 |
| SEM | 0.093 | |  |

Means followed by different uppercase letters within rows and lowercase letters within columns differ (P < 0.05). SEM = standard error of the mean.

**Supplementary Table 11.** Unfolding of the significant interaction between basal diet, phytase, and xylanase supplementation for inositol tetrakisphosphate (InsP4) concentration in the digesta of European quails.

| Phytase - Xylanase | Basal | | *P-value* |
| --- | --- | --- | --- |
|  | Corn | Wheat |  |
| 0 Phytase; 0 Xylanase | 1.09 | 0.84 | 0.062 |
| 0 Phytase; 16000 Xylanase | 0.99 A | 0.63 B | 0.007 |
| 2000 Phytase; 0 Xylanase | 0.89 A | 0.37 B | 0.002 |
| 2000 Phytase; 16000 Xylanase | 0.53 | 0.54 | 0.911 |
| SEM | 0.089 | |  |
| Basal - Xylanase | Phytase | | *P-value* |
|  | 0 | 2000 |  |
| Corn; 0 Xylanase | 1.09 | 0.89 | 0.121 |
| Corn; 16000 Xylanase | 0.99 A | 0.53 B | 0.001 |
| Wheat; 0 Xylanase | 0.84 A | 0.37 B | 0.001 |
| Wheat; 16000 Xylanase | 0.63 | 0.54 | 0.502 |
| SEM | 0.089 | |  |
| Basal - Phytase | Xylanase | | *P-value* |
|  | 0 | 16000 |  |
| Corn; 0 Phytase | 1.09 | 0.99 | 0.434 |
| Corn; 2000 Phytase | 0.89 A | 0.53 B | 0.007 |
| Wheat; 0 Phytase | 0.84 | 0.63 | 0.098 |
| Wheat; 2000 Phytase | 0.37 | 0.54 | 0.183 |
| SEM | 0.089 | |  |

Means followed by different uppercase letters within rows and lowercase letters within columns differ (P < 0.05). SEM = standard error of the mean.

**Supplementary Table 12.** Unfolding of the significant interaction between basal diet, phytase, and xylanase supplementation for inositol trisphosphate (InsP3) concentration in the digesta of European quails.

| Phytase | Basal | | *P-value* |
| --- | --- | --- | --- |
|  | Corn | Wheat |  |
| 0 | 0.45 Ba | 0.71 Aa | 0.001 |
| 2000 | 0.29 Bb | 0.39 Ab | 0.049 |
| *P-value* | 0.002 | 0.001 |  |
| SEM | 0.089 | |  |

Means followed by different uppercase letters within rows and lowercase letters within columns differ (P < 0.05). SEM = standard error of the mean.
